# Primary High-Grade Ovarian Sertoli–Leydig Cell Tumor With Bilateral Adnexal Involvement in a Patient Diagnosed With Peutz–Jeghers Syndrome

**DOI:** 10.1155/crom/3815492

**Published:** 2025-11-19

**Authors:** Tuba Bozhuyuk Sahin, Bayram Burak Ceviz, Ozge Ozmen, Gulay Turan, Figen Efe Camili, Gurhan Guney, Mine Islimye Taskin, Selim Afsar

**Affiliations:** ^1^Department of Obstetrics and Gynecology, Faculty of Medicine, Balikesir University, Balikesir, Turkey; ^2^Department of Pathology, Faculty of Medicine, Balikesir University, Balikesir, Turkey

**Keywords:** adnexal diseases, neoplasm staging, ovarian neoplasms, Peutz–Jeghers syndrome, Sertoli–Leydig cell tumor, sex cord–gonadal stromal tumors

## Abstract

**Aim:**

Peutz–Jeghers syndrome is a rare genetic disease with an increased risk of gastrointestinal and extragastrointestinal malignancies. Ovarian involvement of Sertoli–Leydig cell tumors is uncommon and even more rare in Peutz–Jeghers syndrome patients. This case report outlines the importance of primary ovarian Sertoli–Leydig cell tumor with bilateral adnexal involvement in a patient diagnosed with Peutz–Jeghers syndrome.

**Case:**

A 31-year-old female patient diagnosed with Peutz–Jeghers syndrome presented to our clinic with pelvic pain. Ultrasound examination revealed solid masses in both adnexa. Laparoscopic fertility-preserving surgery was performed. Pathology confirmed the diagnosis of poorly differentiated solid ovarian Sertoli–Leydig cell tumor. Staging surgery was performed, and finally, the patient was referred to oncology for chemotherapy.

**Conclusion:**

This case report outlines the importance of Sertoli–Leydig cell tumors in the differential diagnosis of adnexal masses in patients with Peutz–Jeghers syndrome. Bilateral adnexal involvement is an unusual presentation of Sertoli–Leydig cell tumors. Despite unusual and different presentations, Sertoli–Leydig cell tumors should not be ignored or overlooked in patients with Peutz–Jeghers syndrome.

## 1. Introduction

Peutz–Jeghers syndrome (PJS) is a rare autosomal dominantly inherited disease that is caused by the STK11 gene mutation on chromosome 19p13.3. The three diagnostic criteria for PJS are gastrointestinal polyps, mucocutaneous hyperpigmentation, and family history of PJS. The presence of at least two of these three criteria is required for diagnosis [1–3]. The risk of developing intestinal and extraintestinal cancer is increased in PJS. Cancer development in patients with PJS is particularly common in the small intestine, stomach, pancreas, colon, and esophagus [4]. The genital tract malignancy in women with PJS is underdiagnosed. Cervical gastric-type endocervical adenocarcinoma, endometrial carcinoma, and ovarian neoplasms are among the cancers of the female genital tract. The most common ovarian malignant neoplasms are annular tubular sex cord tumor, Sertoli cell tumor, mucinous epithelial tumor, serous tumor, and mature teratoma [5]. Ovarian neoplasia in women with PJS is rarely reported in the literature. In this report, a case of PJS with primary solid ovarian Sertoli–Leydig cell tumor (SLCT) is presented.

## 2. Case Report

**Figure 1:**
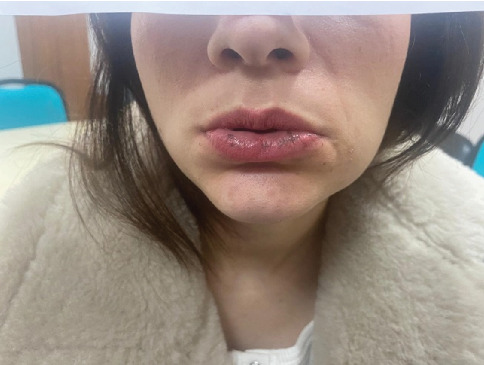
Mucocutaneous hyperpigmentation on the lower lip.

Our 31-year-old patient was diagnosed with PJS at the age of 22. In 2016 and 2019, she underwent abdominal surgery for ileus. The patient has mucocutaneous hyperpigmentations on the lower lip and gingiva (Figure 1) since her childhood. She was first diagnosed with PJS after the identification of hamartomatous polyps in a colonoscopic biopsy. After her diagnosis, she has undergone regular endoscopic and colonoscopic polypectomy every 3 years. The patient's daughter was also diagnosed with PJS at the age of three.

The patient presented to our outpatient clinic with pelvic pain. On the transvaginal ultrasound examination, she was diagnosed with a multilobulated, irregular-bordered solid cyst with a diameter of 5 cm in the left adnexa. Additionally, hydrosalpinx was observed on the left fallopian tube. In the right adnexa, a solid, regular-bordered cyst with a diameter of 4 cm was detected. The CA-125 level was 46 U/mL, showing a minimally increased level.

**Figure 2:**
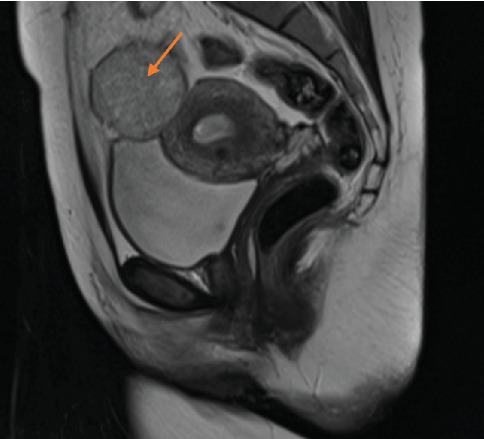
Mass on the left adnexa (MR sagittal section) (orange arrow).

**Figure 3:**
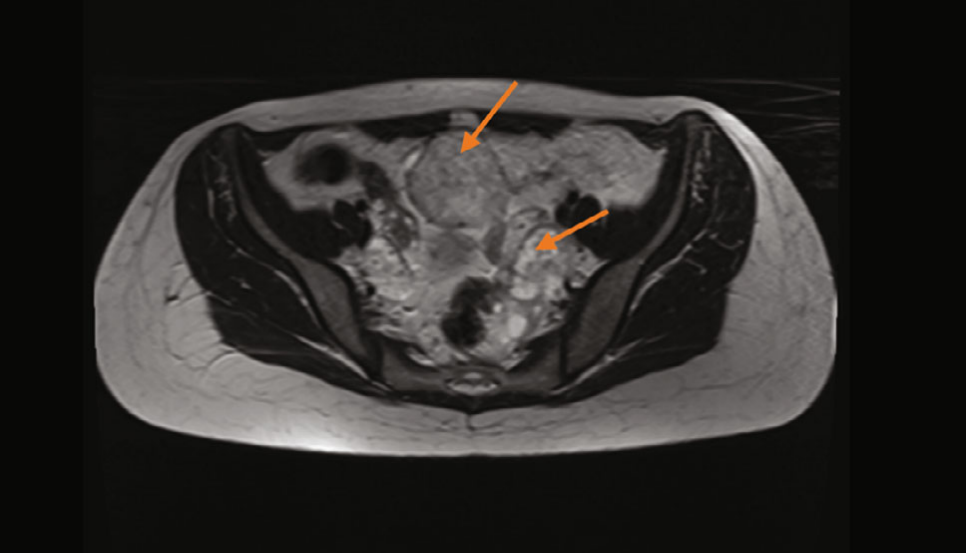
Mass on the left adnexa (MR transverse section) (orange arrows).

**Figure 4:**
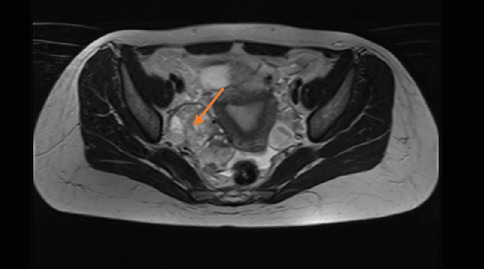
Mass on the right adnexa (MR transverse section) (orange arrow).

Preoperative magnetic resonance imaging (MRI) revealed a 5-cm heterogeneously enhancing mass lesion on the left adnexa and a 4-cm mass on the right adnexa (Figures 2, 3, and 4).

**Figure 5:**
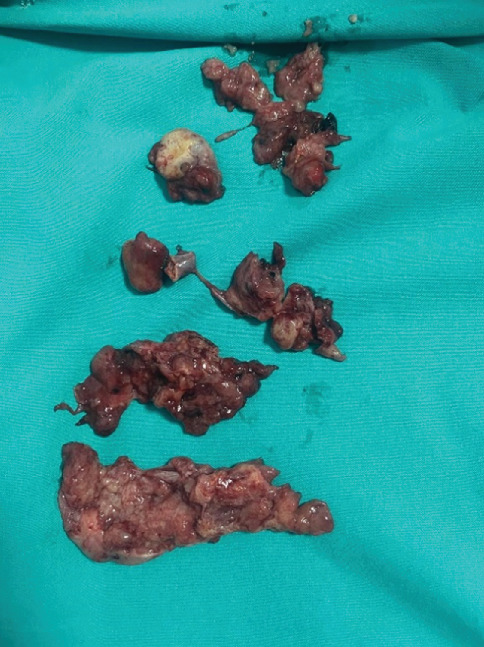
Masses removed from the left adnexa.

**Figure 6:**
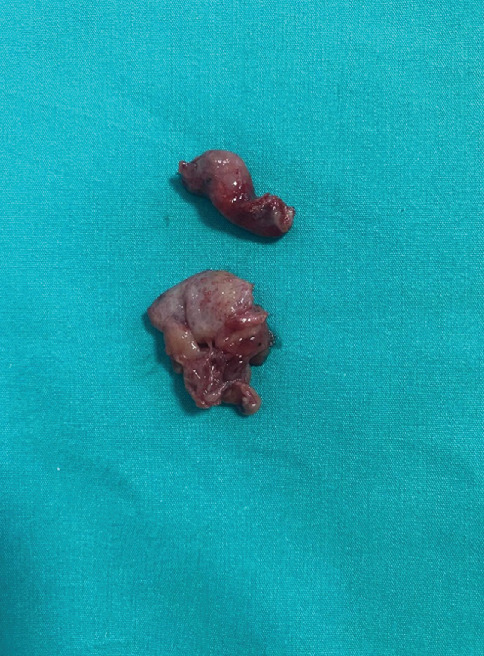
Left tube on the top and mass removed from the right adnexa on the bottom.

During diagnostic laparoscopy, the uterus size and the anterior–lateral serosal wall appeared normal. A multilobulated, atypical solid cyst with a diameter of 5 cm was identified on the left adnexal side and another solid cyst measuring 4 cm was found on the right adnexal side. Both cysts were located on the uterosacral ligaments separated from the ovaries. Hydrosalpinx was present in both fallopian tubes. As the patient desired to preserve fertility, she requested to retain one of her tubes. Therefore, the right fallopian tube was preserved. Ultimately, only the bilateral adnexal masses and the left fallopian tube were removed (Figures 5 and 6), while the uterus, bilateral ovaries, and right fallopian tube were preserved. No pathological findings were observed on the pelvic peritoneum.

**Figure 7:**
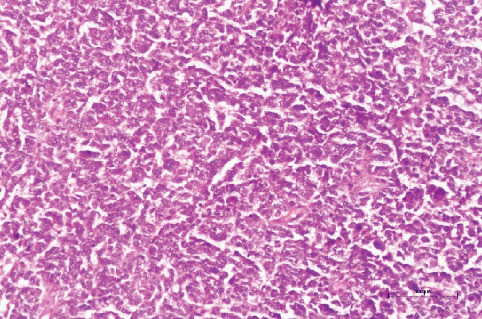
Diffuse pattern, small round nuclei, and columnar cells with pale eosinophilic cytoplasm (hematoxylin and eosin, 200×).

**Figure 8:**
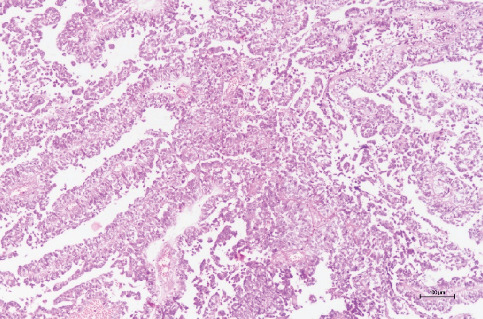
Tumor cells exhibiting a pseudopapillary configuration (hematoxylin and eosin, 100×).

**Figure 9:**
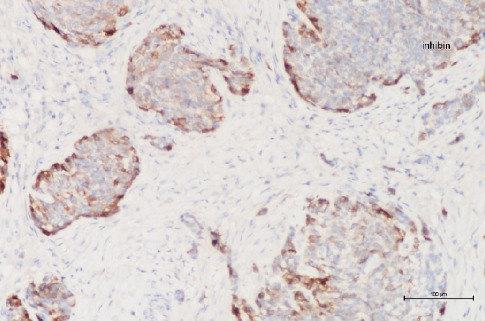
In immunohistochemical analysis, positive staining with inhibin is observed in Sertoli cell tumor (100×).

**Figure 10:**
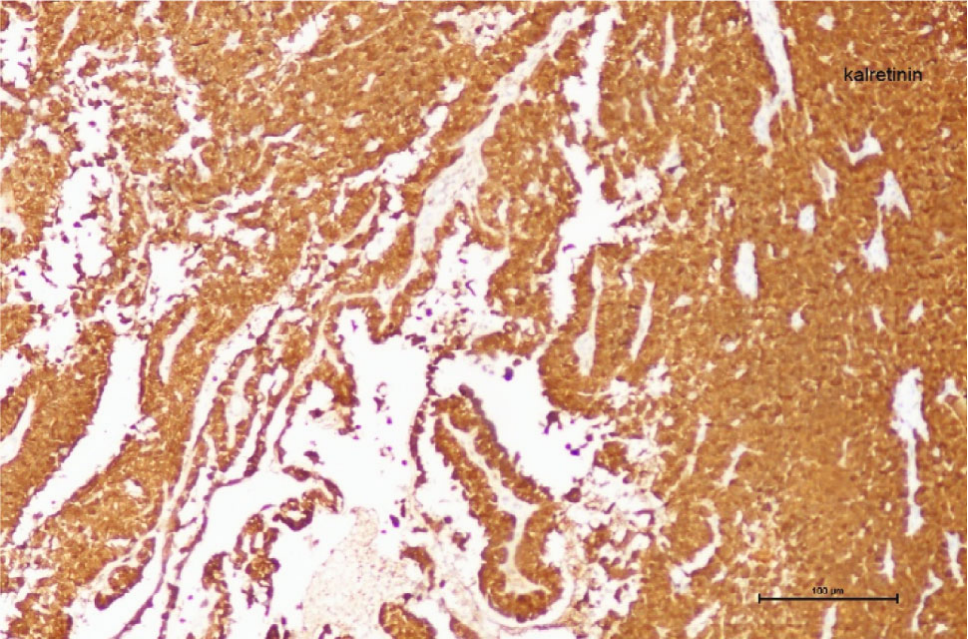
In immunohistochemical analysis, positive staining with calretinin is observed in Sertoli cell tumor (100×).

**Figure 11:**
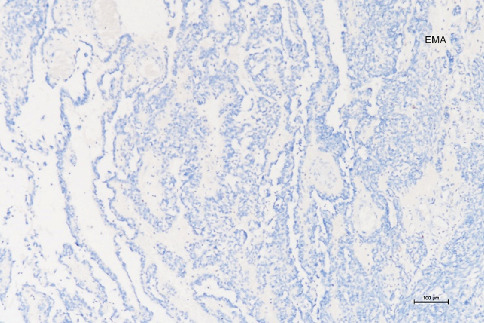
Immunohistochemically, EMA negativity is important in distinguishing between epithelial neoplasms and has been interpreted in favor of a sex cord–stromal tumor (100×).

The pathology report confirmed the diagnosis of poorly differentiated solid ovarian SLCT (Figures 7, 8, 9, 10, and 11).

**Figure 12:**
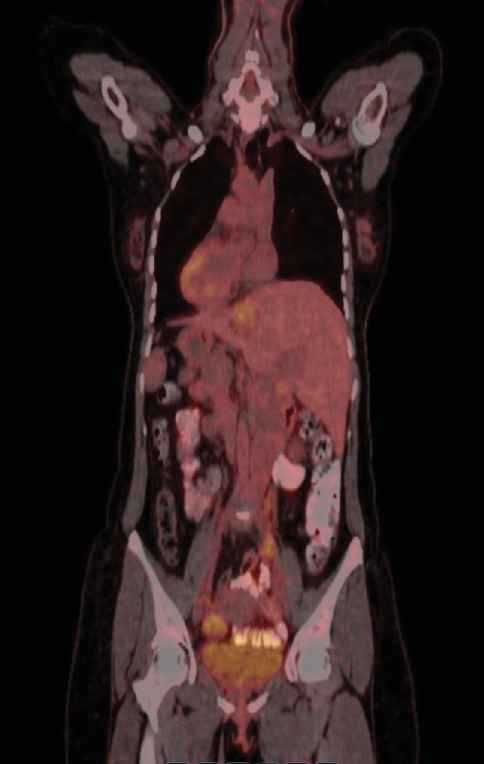
PET coronal section.

**Figure 13:**
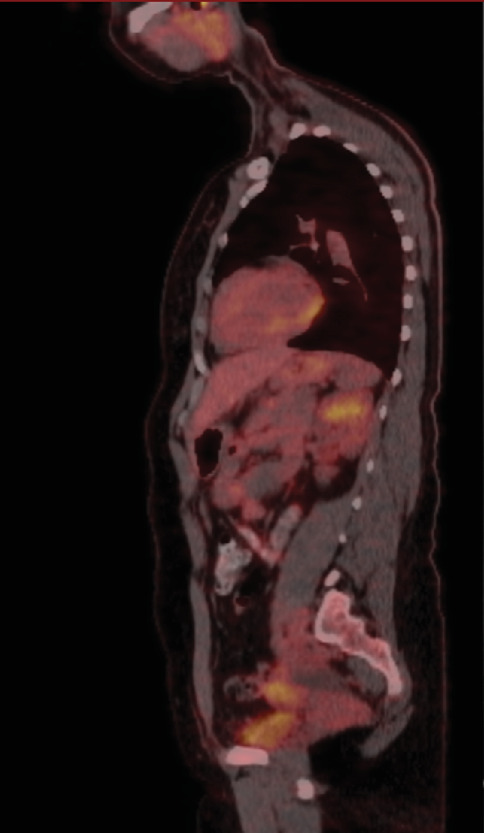
PET sagittal section.

**Figure 14:**
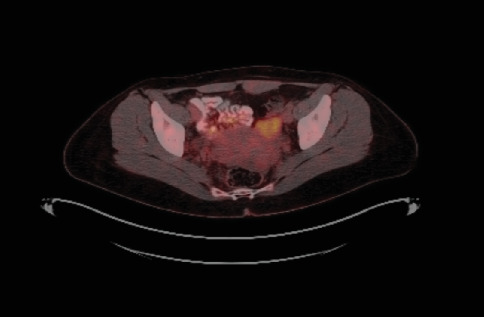
PET transverse section.

Positron emission tomography (PET) imaging after the operation showed a hypermetabolic focus in the left ovary, consistent with primary malignancy. Additionally, a semisolid hypermetabolic focus was observed in the right ovary (Figures 12, 13, and 14).

Staging surgery was performed. Laparoscopic observation revealed a normal uterus. Since the left ovary was identified as the primary focus of the malignancy, a left oophorectomy was performed. Additionally, a right salpingectomy was carried out. Implants on the left bladder and sigmoid colon were removed. Furthermore, bilateral pelvic lymph node dissection and total omentectomy were performed.

The pathologies of the implants and lymph nodes we received were again consistent with SLCT infiltration. Finally, the patient was referred to medical oncology for chemotherapy. No pathological findings were detected on the postoperative MRI, CT, and PET-CT after two cycles of chemotherapy.

## 3. Discussion

SLCT is a subtype of sex cord–stromal tumors and creates less than 0.5% of all ovarian neoplasms. This risk rises to 20% in PJS patients [6, 7]. Despite its rarity, it is clinically significant, as its prognosis and management depend on tumor differentiation and stage at diagnosis [8]. SLCTs can occur at any age. Seventy-five percent of patients are affected at < 30 years while 10% of the patients are > 50 years old [9]. Our patient is 31 years old, which is within the typical age range. However, in 2016, Bellfield et al. reported a case of recurrent SLCT in a child with PJS at the ages of 3 and 17, which shows that SLCT can occur at any age and may even recur [10].

SLCTs exhibit symptoms similar to other ovarian tumors, including abdominal and pelvic pain, distention, amenorrhea, oligomenorrhea, and virilization [6]. The patient in our case presented with the typical symptoms of abdominal and pelvic pain. However, menstrual dysfunction and signs of virilization were not observed. Additionally, the blood testosterone levels were normal. In contrast, Mudraje et al. reported a 30-year-old woman with androgenic alopecia, virilization, secondary amenorrhea, and extremely high testosterone levels [11]. The differences between these two case presentations underscore the wide variability in the clinical manifestations of SLCT. SLCTs are highly heterogeneous in terms of their presentation, hormonal activity, and clinical outcomes.

SLCTs are generally solid, lobulated tumors of various sizes [12]. They are typically unilateral, with sizes ranging from less than 1 to 51 cm (mean size of 13.5 cm) [13]. The tumor in our case was also solid and lobulated. However, our case deviated from the standard because of its presentation on both adnexal sides with a diameter of 5 cm on the left and 4 cm on the right. Poorly differentiated SLCTs tend to be larger, edematous, hemorrhagic, and necrotic [14]. Correspondingly, the tumor mass in our case exhibited atypical characteristics and necrosis.

SLCTs are uncommon ovarian neoplasms, and they are even more rare in PJS patients. This case report outlines the importance of SLCTs in the differential diagnosis of adnexal masses in patients with PJS. Bilateral adnexal involvement, as observed in our patient, is an unusual presentation of SLCTs. Despite unusual and different presentations, SLCTs should not be ignored or overlooked in patients with PJS. The early diagnosis of this tumor and its appropriate surgical management are important for optimizing clinical outcomes. This aspect is especially important for young women desiring fertility preservation. Additionally, there are currently no established guidelines for screening relatives and children of patients with PJS. Genetic screening and family counseling should be offered to the relatives of PJS patients for the early detection of SLCTs and other ovarian tumors. In conclusion, this case report emphasizes that malignant ovarian involvement should not be overlooked in patients with PJS.

## Data Availability

The data used in this case report may be provided upon reasonable request to the relevant authors.
